# Hypericin in the Dark: Foe or Ally in Photodynamic Therapy?

**DOI:** 10.3390/cancers8100093

**Published:** 2016-10-14

**Authors:** Veronika Huntosova, Katarina Stroffekova

**Affiliations:** 1 Center for Interdisciplinary Biosciences, PJ Safarik University in Kosice, Kosice 040 01, Slovakia; veronika.huntosova@upjs.sk; 2 Department of Biophysics, Faculty of Natural Sciences, PJ Safarik University in Kosice, Kosice 040 01, Slovakia

**Keywords:** hypericin, PKC, Bcl2, BAX, oxidative stress, glioma cells, endothelial cells, cancer

## Abstract

Photosensitizers (PSs) in photodynamic therapy (PDT) are, in most cases, administered systemically with preferential accumulation in malignant tissues; however, exposure of non-malignant tissues to PS may also be clinically relevant, when PS molecules affect the pro-apoptotic cascade without illumination. Hypericin (Hyp) as PS and its derivatives have long been studied, regarding their photodynamic and photocytotoxic characteristics. Hyp and its derivatives have displayed light-activated antiproliferative and cytotoxic effects in many tumor cell lines without cytotoxicity in the dark. However, light-independent effects of Hyp have emerged. Contrary to the acclaimed Hyp minimal dark cytotoxicity and preferential accumulation in tumor cells, it was recently been shown that non-malignant and malignant cells uptake Hyp at a similar level. In addition, Hyp has displayed light-independent toxicity and anti-proliferative effects in a wide range of concentrations. There are multiple mechanisms underlying Hyp light-independent effects, and we are still missing many details about them. In this paper, we focus on Hyp light-independent effects at several sub-cellular levels—protein distribution and synthesis, organelle ultrastructure and function, and Hyp light-independent effects regarding reactive oxygen species (ROS). We summarize work from our laboratories and that of others to reveal an intricate network of the Hyp light-independent effects. We propose a schematic model of pro- and anti-apoptotic protein dynamics between cell organelles due to Hyp presence without illumination. Based on our model, Hyp can be explored as an adjuvant therapeutic drug in combination with chemo- or radiation cancer therapy.

## 1. Introduction

Photodynamic therapy (PDT) is now widely used for diagnostic and treatment purposes in case of cancer, inflammatory diseases, infections, and stimulating antitumor immunity [1,2,3]. Photosensitizers (PSs) used in PDT are usually administered systemically and show a preferential accumulation in malignant cells. Nevertheless, exposure of healthy non-malignant cells to PS remains potentially high, especially in the vasculature [4]. Tumor response to PDT is variable, ranging from high sensitivity to extreme resistance. The parameters determining tumor sensitivity to PDT are PS cellular distribution, tumor oxygenation, vascularity, and immunogenicity [5]. 

Hyp and its derivatives have been used in experimental PDT approaches for a long time [2,3,6,7]. They have displayed illumination-dependent, anti-proliferative, and cytotoxic effects in many tumor cell lines [8,9]. The type of HypPDT triggered cell death signaling pathway (apoptosis or necrosis) is determined by the sub-cellular Hyp accumulation sites in membranous organelles [7,9,10].

PDT can result in apoptosis via multiple signaling pathways including death receptors, caspases, Bcl2 family proteins, and mitochondrial dysfunction.

Protein kinase C (PKC) comprises a large family of Ser/Threonine (Thr) kinases that play a fundamental role in many physiological processes, including the immune response, cell proliferation and differentiation, and apoptosis [11,12]. The PKC family consists of at least 10 subtypes that can be classified into three subgroups classical, novel, and atypical [13]. The specific PKC isoforms can be either anti- or pro-apoptotic depending on cell and stimulus type [14]. In various cell types including U87 MG, PKCα, and PKCδ isotypes exhibit an opposing effect in the cell survival and apoptosis [12,15,16,17]. A pro-apoptotic PKCδ has been shown to be a target of caspase-3, where anti-apoptotic PKCα inhibits apoptosis by phosphorylating Bcl2 [11,17].

Bcl2 and other members of the Bcl2 family of proteins play a key role in the regulation of apoptosis. Pro- and anti-apoptotic members of the Bcl2 family control cell survival or death response through protein-protein interactions [18]. Originally, members of the Bcl2 family were thought to be present in the cytoplasm and mitochondria. In the last decade, they have also been shown to be present in the endoplasmic reticulum (ER), Golgi apparatus (GA), and nucleus. Members of the Bcl2 family participate in apoptosis regulation either at mitochondria or by the regulating of ER Ca^2+^ homeostasis [19,20]. 

The PS Hyp has been claimed to display a minimal dark (light-independent) cytotoxicity [6,7,9], however, recently it was shown that Hyp had light-independent cytotoxic effects in a wide range of concentrations in various cell types [4,21,22]. The Hyp light-independent effects could be due to various mechanism, for example reduction of intracellular pH, or due to inhibition of different enzymes [4,23,24,25]. There is also possibility that multiple mechanisms are running simultaneously, due to Hyp interaction with different molecules at the Hyp accumulation sites such as mitochondria, ER and GA. 

Here, we are focusing on Hyp light-independent effects at several sub-cellular levels—protein distribution and synthesis, organelle ultrastructure and function, and Hyp light-independent effects regarding ROS. Up to this date, findings regarding Hyp light-independent effects are as follows. (I) Hyp colocalizes with PKCα in U87 MG cells, and Hyp binding assays and molecular modeling indicate direct interactions between Hyp and PKCα [26,27]; (II) in U87 MG cells, the majority of phosphorylated Bcl2 co-localizes with PKCα [25]; (III) Hyp in the dark follows the ceramide pathway, translocates, and increases PKCδ autophosphorylation at Ser645 in the GA and nucleus of U87 MG cells [28]; (IV) Hyp affects distribution of pro-apoptotic proteins Bak and Bax, and anti-apoptotic Bcl2 in human glioma U87 MG and endothelial HCAEC cells [29,30]; (V) Hyp affects cell viability in metabolically distinct cell phenotypes differently [4,21,22,25,30]. In addition, our newest work suggests that Hyp causes the light-independent effects in bioenergetics, increases oxidative stress, and results in adaptive changes in ultrastructure.

Here, we will summarize work from our laboratories and that of others to reveal an intricate network of the Hyp light-independent effects. We propose a schematic model of pro- and anti-apoptotic protein dynamics between cell organelles due to Hyp presence without illumination. Based on our model, Hyp can be explored as adjuvant therapeutic drug in combination with chemo- or radiation cancer therapy.

## 2. Hyp Light-Independent Effects on the Members of Bcl2 Family of Proteins

**Figure 1:**
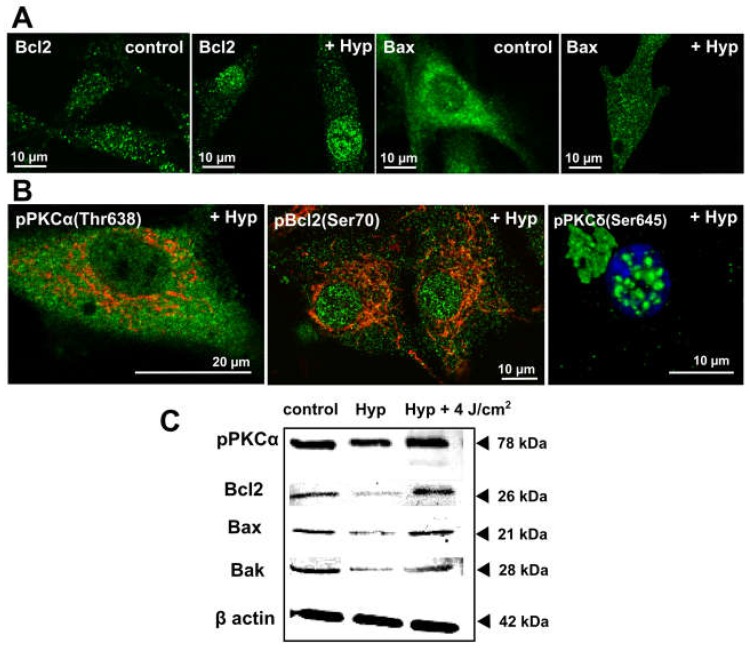
The dark Hyp effects on the distribution of Bcl2 family and PKC family members. (**A**) Representative fluorescence images of ice-cold methanol fixed U87 MG cells treated for 1 h with 500 nM of Hyp in the dark. Cells were immunostained with antibodies against Bcl2 (**green**), Bax (**green**). (**B**) PKCα phosphorylated on Thr638 (**green**), Bcl2 phosphorylated on Ser70 (**green**), and PKCδ phosphorylated on Ser645 (**green**). The mitochondria are stained with TMROS Orange (**red**) and nucleus with Hoechst 33342 (**blue**). The fluorescence images were acquired by LSM700 confocal microscope (Zeiss, Germany) with 63X oil objective (NA = 1.46). The fluorophores were excited and detected under these conditions: Hoechst (405/410–490 nm), Alexa 488 conjugated with secondary antibodies for visualization of Bcl2, PKCα and PKCδ (488/500–550 nm), MitoTracker® Orange CMTM/Ros (555/590–630 nm). (**C**) The corresponding Western blot analysis results of selected proteins in U87 MG cells with and without Hyp treatment in the dark, or 1 h after irradiation (4 J/cm^2^). The experimental protocols are described in detail in previous publications [25,28,30].

Members of the Bcl2 family of proteins localize at mitochondria and sites of ER and mitochondria contacts [19,20,31,32]. They play a key role in mitochondria apoptotic pathway and were shown to regulate mitochondria morphogenesis (fission/fusion) [19,20,33,34]. We studied the Hyp light-independent effects on members’ distribution of the Bcl2 family in malignant U87 MG and non-malignant HCAEC cells [29,30]. The U87 MG is a commercially available grade IV human glioma cell line, which was used for various research purposes over four decades. The HCAEC are commercially available primary Human Coronary Artery Endothelial Cells isolated from the coronary arteries from a healthy donor. Hyp (500 nM) in U87 MG and HCAEC cells significantly changed the distribution of Bcl2 and Bax proteins (Figure 1A).

In both cell types under control conditions, the majority of Bcl2 localized in discrete foci, presumably mitochondria [29] and the ER and mitochondria contact sites. Incubation with Hyp changed Bcl2 distribution (Figure 1A). The Bcl2 signal apparently decreased in foci outside the nucleus, and there was noticeable Bcl2 translocation into nuclei in U87 MG (Figure 1) and HAEC cells [30]. Bcl2 translocation into nuclei can enable the on-set of apoptosis via the association with nuclear receptor Nur77, which plays a role in regulation of differentiation, proliferation, apoptosis, and survival in different cell types [35].

Under control conditions in both cell types, Bax distribution showed a diffused pattern with some localization in foci, corresponding to distributions in many cell types [31,32,33]. Hyp presence resulted in distinctive changes in Bax distribution. In U87 MG (Figure 1A) and HCAEC [30], there was significant Bax translocation into distinct foci throughout the cell. In HCAEC, there was also a substantial Bax translocation into the nuclei. Bax translocation to mitochondria is often associated with an apoptosis, whereas translocation to the nucleus/ER has been indicated with necrosis, inflammation, and secondary apoptosis [36,37]. The Hyp light-independent effects on the Bcl2 proteins distribution are dependent on the actual intracellular concentration of Hyp. The intracellular Hyp concentration depends on the final concentration of Hyp in the cell media, incubation times and on the cell uptake rate [38,39]. Bcl2 and Bax translocation patterns in U87 MG and HCAEC cells due to Hyp in the dark may underlie a different cell response to Hyp in viability assays measured via flow cytometry with Annexin V and propidium iodide [30,39]. In U87 MG, Hyp did not affect cell viability [25,29], and in the HCAEC resulted in a significant decrease in viability from 90% to 50% [30]. Besides the translocation of Bcl2 and Bax, Hyp light-independent treatment also caused a decrease in the protein synthesis, as indicated by the Western blot analysis (Figure 1C) [25].

The difference may be due to either different Bax distribution changes in U87 MG and HAEC (nucleus vs ER) or the fact that malignant U87 MG cells have additional survival mechanisms in response to Hyp, such as Bcl2 interaction with DNA repair protein Ku70 [40,41]. In addition, we have shown that the majority of Bcl2 protein present in U87 MG cells is phosphorylated at serine 70 (pBcl2S70) [25]. It has been shown that phosphorylation of Bcl2 at serine 70 is required for Bcl2’s full and potent anti-apoptotic function [11,12]. The distribution of Bcl2 in the presence of Hyp (Figure 1A) follows the same pattern as pBcl2S70 [25].

## 3. Hyp Light-Independent Effects on the Distribution and Phosphorylation of Anti-Apoptotic PKCα and Pro-Apoptotic PKCδ

In various cell types including U87 MG, PKCα, and PKCδ isotypes exhibit an opposing effect in cell survival and apoptosis [12,15,16,17]. A pro-apoptotic PKCδ has been shown to be a target of caspase-3, where anti-apoptotic PKCα inhibits apoptosis by phosphorylating Bcl2 [11,17]. Generally, inactive PKCs are considered to be cytoplasmic; upon activation by different signals, PKC translocate to the plasma membrane, to other membraneous organelles, and to the nucleus [11,42].

We have shown that the majority of PKCα present in U87 MG cells is already in a catalytically competent phosphorylated form pPKCα(Thr638) (Figure 1B; and in [25]). This was in agreement with published works regarding the increased activity of PKCα in gliomas and glioma cell lines [11,12]. In addition, we have shown that the majority of pBcl2S70 protein present in U87 MG cells co-localizes with PKCα [25], suggesting that PKCα is likely one of the Bcl2 kinases in U87 MG cells. 

**Figure 2:**
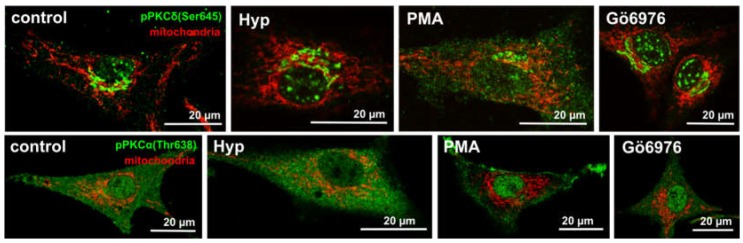
The mitochondria (**red**) and pPKCα(Thr638) (**green**) or pPKCδ(Ser645) (**green**) distribution in U87 MG cells non-treated (control) or treated for 1 h with either 500 nM of Hyp, or 100 nM of PMA (PKCα activator) and 100 nM of Gö6976 (PKCα inhibitor), which also influences pre-mature PKCδ localization (pPKCδ(Ser645)) [25,28].

Hyp was shown to co-localize with PKCα in U87 MG cells, and Hyp binding assays and molecular modeling indicated direct interactions between Hyp and PKCα [26,27]; however, Hyp presence did not affect PKCα distribution in U87 MG cells [25]. In the control cells and in the cells treated with Hyp without irradiation, the majority of PKCα was distributed evenly in the cytoplasm and at mitochondria (Figure 1B; and in [25]). However, we have also shown that pretreatment with Hyp decreased PKCα translocation to the plasma membrane upon PMA treatment (Figure 2 vs. Figure 6 in Dzurova et al. [25]) and increased cytoplasmic localization of PKCα. This finding suggests that Hyp competes for the PMA binding site at PKCα and prevents PKCα activation [25]. Dissociation constant K_d_ for the Hyp binding to PKCα and to PKCδ was determined to be 111 nM and 94 nM, respectively [27]. These values are slightly lower than the binding of PMA (K_d_ = 160 nM) for both isoforms, which reflects that Hyp can be a strong competitor with phorbol esters for the binding to these PKC isoforms [27]. Thus, we also investigated Hyp light-independent effects on the pro-apoptotic PKCδ distribution and phosphorylation in U87 MG cells [25,28]. 

We have shown the distribution of PKCδ and its phosphorylated form pPKCδ(S645) in the absence and the presence of the Hyp [28]. The S645 is considered a priming phosphorylation site, and it has been suggested that this modification converts PKCδ to its mature form [28,42,43]. There was no difference in non-specific PKCδ staining. Figure 2 shows a comparison of primed phosphorylated PKCα (pPKCα (Thr638)) and PKCδ(pPKCδ(S645)) forms in the presence of PKC activator (PMA), inhibitor (Gö6976) and Hyp, respectively [28]. The light-independent effect of Hyp on p(S645)PKCδ is similar to the PKC inhibitor Gö6976. In addition, we have identified that the p(S645)PKCδ signal localization in the presence of Hyp and Gö6976 is most likely GA-related compartments. Further, it was shown that Hyp is likely to follow the same ceramide’s uptake pathway [28].

The cleavage of PKCδ and the accumulation of the constitutively active fragment in the nuclei and in the Golgi apparatus (GA) are critical for triggering the nuclear fragmentation and for the ceramide-induced apoptosis [15,17].

It has been shown that PKCα activity blocks the ROS production and, in return, that the Gö6976 inhibitor treatment is triggering a rapid increase in ROS in the mitochondria [14]. Mitochondria play an integrative role in controlling cell ROS production [44,45]. Further, it was also shown that, besides its anti-apoptotic activity, Bcl2 overexpression could protect the cells from the oxidative stress induced by a variety of oxidative insults [46].

**Figure 3:**
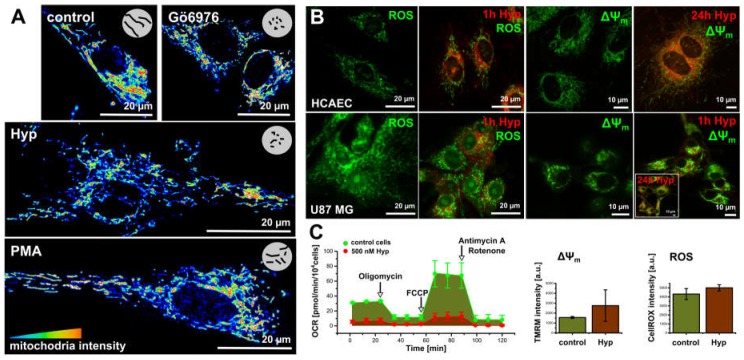
The dark Hyp effects on the mitochondria morphogenesis and oxidative stress. (**A**) The mitochondria morphology stained with MitoTracker Orange in 16 colors representation in the methanol fixed cells: control cells and in cells treated for 1 h with either 500 nM of Hyp, or 100 nM of PMA and 100 nM of Gö6976. (**B**) ROS (CellROX **green**) and mitochondrial potential (∆ψ_m_, Rhodamin 123, or TMRM) in lived HCAEC and U87 MG cells. Signals were acquired in control cells and in cells treated with Hyp for 1 h or 24 h in the dark. (**C**) The metabolic profile of U87 MG cells in the absence (**green**) and in the presence of Hyp (**red**). The profiles were measured with an extracellular flux analyzer Seahorse XF24. The flow cytometry histograms represented the increase of ∆ψ_m_ and ROS in cells with and without Hyp. The experimental protocols are described in detail in other publications [25,28,30].

In light of these facts, we investigated Hyp light-independent effects on the oxidative stress, and mitochondria structure and function (Figure 3).

## 4. Hyp Light-Independent Effects on the Mitochondria Ultrastructure and Function

To better understand the Hyp light-independent effects, we investigated the Hyp effect on mitochondrial function and cell metabolism in malignant U87 MG and non-malignant HCAEC cells.

To monitor the Hyp effect on the mitochondria structure, we used organelle specific fluorescent dye MitoTracker Orange in the presence and absence of Hyp, PMA, and Gö6976 inhibitor (Figure 3A) in the methanol fixed cells. Control cells and cells treated with PMA show a widespread mitochondrion network, which is also evident in the live cells stained with mitochondrial potential (ΔΨ_m_) indicator Rhodamine123 (Figure 3B). The presence of either Hyp or Gö6976 causes slight fragmentation of the mitochondrion (Figure 3A).

In addition to slight fragmentation, the presence of Hyp also results in the ROS increase in both cell lines (Figure 3B,C). However, the increase in ROS is not due to dissipation of the mitochondrial potential (ΔΨ_m_). In contrast, Hyp results in slight hyperpolarization as it is indicated in Figure 3B by the increase in the Rho123 fluorescence intensity (Figure 3B,C).

To examine the Hyp effects on the mitochondrial function, we investigated the cellular bioenergetics in intact U87 MG (Figure 3C) and HCAEC cells [30]. A detailed description of the method and protocols are in [30]. Figure 3C shows the oxygen consumption rate (OCR) measurements in U87 MG cells. Bioenergetics profiles reflect the U87 MG and HCAEC high proliferation and metabolic rates, respectively. U87 MG characteristics indicate a high proliferation and metabolic rates, and that substantial energy proportion originates from oxidative phosphorylation (OXPHOS) in addition to glycolysis. HCAEC characteristics indicates low proliferation and metabolic rates, and glycolysis as a dominant energy source [30].

Hyp significantly influenced the metabolism of U87 MG cells (Figure 3C). U87 MG respiration significantly decreased, and Hyp also resulted in a proton leak decrease, which is in agreement with the observed hyperpolarization of mitochondrial potential (Figure 3B,C). The Hyp presence in U87 MG cells seems to slow down overall cell metabolism, OXPHOS, and glycolysis. Hyp presence did not change overall the OCR profile in HCAEC, reflecting the glycolysis as a dominant energy source.

**Figure 4:**
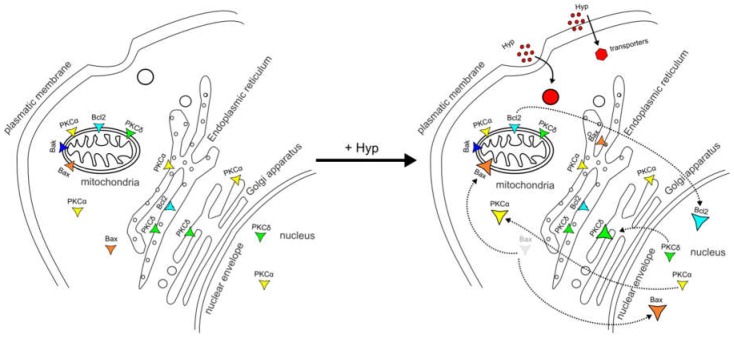
Schematic model of pro- and anti-apoptotic protein dynamics between the organelles after Hyp administration.

Based on work from our laboratories and that of others, we propose a schematic model of pro- and anti-apoptotic protein dynamics between cell organelles due to Hyp presence without illumination (Figure 4). Based on our model, Hyp can be explored as an adjuvant therapeutic drug in combination with chemo- or radiation cancer therapy.

## 5. Conclusions

In conclusion, we have shown that Hyp has significant light-independent effects in malignant and non-malignant cells at several sub-cellular levels such as protein synthesis and distribution (Bcl2 family, PKC), mitochondria structure and function, and Hyp light-independent effects regarding ROS. Our findings suggest that Hyp without illumination can be explored as adjuvant therapeutic drug in combination with chemo- or radiation cancer therapy.

## References

[1] Bechet D., Mordon S.R., Guillemin F., Barberi-Heyob M.A. (2014). Photodynamic therapy of malignant brain tumours: A complementary approach to conventional therapies. Cancer Treat. Rev..

[2] Berlanda J., Kiesslich T., Engelhardt V., Krammer B., Plaetzer K. (2010). Comparative in vitro study on the characteristics of different photosensitizers employed in PDT. J. Photochem. Photobiol. B.

[3] Kepp O., Senovilla L., Vitale I., Vacchelli E., Adjemian S., Agostinis P., Apetoh L., Aranda F., Barnaba V., Bloy N. (2014). Consensus guidelines for the detection of immunogenic cell death. Oncoimmunology.

[4] Gyenge E.B., Forny P., Luscher D., Laass A., Walt H., Maake C. (2012). Effects of hypericin and a chlorin based photosensitizer alone or in combination in squamous cell carcinoma cells in the dark. Photodiagn. Photodyn. Ther..

[5] Reeves K.J., Reed M.W.R., Brown N.J. (2009). Is nitric oxide important in photodynamic therapy?. J. Photochem. Photobiol. B Biol..

[6] Miskovsky P. (2002). Hypericin—A new antiviral and antitumor photosensitizer: Mechanism of action and interaction with biological macromolecules. Curr. Drug Targets.

[7] Theodossiou T.A., Hothersall J.S., de Witte P.A., Pantos A., Agostinis P. (2009). The multifaceted photocytotoxic profile of hypericin. Mol. Pharm..

[8] Almeida R.D., Manadas B.J., Carvalho A.P., Duarte C.B. (2004). Intracellular signaling mechanisms in photodynamic therapy. Biochim. Biophys. Acta Rev. Cancer.

[9] Krammer B., Verwanger T. (2012). Molecular response to hypericin-induced photodamage. Curr. Med. Chem..

[10] Buytaert E., Callewaert G., Hendrickx N., Scorrano L., Hartmann D., Missiaen L., Vandenheede J.R., Heirman I., Grooten J., Agostinis P. (2006). Role of endoplasmic reticulum depletion and multidomain proapoptotic bax and bak proteins in shaping cell death after hypericin-mediated photodynamic therapy. FASEB J..

[11] Ruvolo P.P., Deng X.M., Carr B.H., May W.S. (1998). A functional role for mitochondrial protein kinase C alpha in Bcl2 phosphorylation and suppression of apoptosis. J. Biol. Chem..

[12] Mandil R., Ashkenazi E., Blass M., Kronfeld I., Kazimirsky G., Rosenthal G., Umansky F., Lorenzo P.S., Blumberg P.M., Brodie C. (2001). Protein kinase C alpha and protein kinase C delta play opposite roles in the proliferation and apoptosis of glioma cells. Cancer Res..

[13] Nishizuka Y. (1995). Protein kinases 5. Protein-kinase-C and lipid signaling for sustained cellular-responses. FASEB J..

[14] Lee S.K., Shehzad A., Jung J.C., Sonn J.K., Lee J.T., Park J.W., Lee Y.S. (2012). Protein kinase calpha protects against multidrug resistance in human colon cancer cells. Mol. Cells.

[15] Halder K., Banerjee S., Bose A., Majumder S., Majumdar S. (2014). Overexpressed pkc delta downregulates the expression of PKC alpha in B16F10 melanoma: Induction of apoptosis by PKC delta via ceramide generation. PLoS ONE.

[16] Berdiev B.K., Xia J.Z., Jovov B., Markert J.M., Mapstone T.B., Gillespie G.Y., Fuller C.M., Bubien J.K., Benos D.J. (2002). Protein kinase C isoform antagonism controls BNAC2 (ASIC1) function. J. Biol. Chem..

[17] Kajimoto T., Shirai Y., Sakai N., Yamamoto T., Matsuzaki H., Kikkawa U., Saito N. (2004). Ceramide-induced apoptosis by translocation, phosphorylation, and activation of protein kinase C delta in the golgi complex. J. Biol. Chem..

[18] Petros A.M., Olejniczak E.T., Fesik S.W. (2004). Structural biology of the Bcl-2 family of proteins. Biochim. Biophys. Acta Mol. Cell Res..

[19] Lindsay J., Esposti M.D., Gilmore A.P. (2011). Bcl-2 proteins and mitochondria—Specificity in membrane targeting for death. Biochim. Biophys. Acta Mol. Cell Res..

[20] Bonneau B., Prudent J., Popgeorgiev N., Gillet G. (2013). Non-apoptotic roles of Bcl-2 family: The calcium connection. Biochim. Biophys. Acta Mol. Cell Res..

[21] Blank M., Lavie G., Mandel M., Hazan S., Orenstein A., Meruelo D., Keisari Y. (2004). Antimetastatic activity of the photodynamic agent hypericin in the dark. Int. J. Cancer.

[22] Martinez-Poveda B., Quesada A.R., Medina M.A. (2005). Hypericin in the dark inhibits key steps of angiogenesis in vitro. Eur. J. Pharmacol..

[23] Sureau F., Miskovsky P., Chinsky L., Turpin P.Y. (1996). Hypericin-induced cell photosensitization involves an intracellular ph decrease. J. Am. Chem. Soc..

[24] Haimovitz-Friedman A., Balaban N., McLoughlin M., Ehleiter D., Michaeli J., Vlodavsky I., Fuks Z. (1994). Protein kinase C mediates basic fibroblast growth factor protection of endothelial cells against radiation-induced apoptosis. Cancer Res..

[25] Dzurová L., Petrovajova D., Nadova Z., Huntosova V., Miskovsky P., Stroffekova K. (2014). The role of anti-apoptotic protein kinase Cα in response to hypericin photodynamic therapy in U-87 mg cells. Photodiagn. Photodyn. Ther..

[26] Kocanova S., Mateasik A., Chorvat D., Miskovsky R. (2005). Multispectral confocal fluorescence imaging: Monitoring of intracellular distribution of PKC influenced by photoactive drug hypericin. Laser Phys. Lett..

[27] Kocanova S., Hornakova T., Hritz J., Jancura D., Chorvat D., Mateasik A., Ulicny J., Refregiers M., Maurizot J.C., Miskovsky P. (2006). Characterization of the interaction of hypericin with protein kinase C in U-87 mg human glioma cells. Photochem. Photobiol..

[28] Misuth M., Joniova J., Belej D., Hrivnak S., Horvath D., Huntosova V. (2016). Estimation of PKCδ autophosphorylation in U87 mg glioma cells: Combination of experimental, conceptual and numerical approaches. J. Biophotonics.

[29] Balogova L., Maslanakova M., Dzurova L., Miskovsky P., Stroffekova K. (2013). Bcl-2 proapoptotic proteins distribution in U-87 mg glioma cells before and after hypericin photodynamic action. Gen. Physiol. Biophys..

[30] Maslanakova M., Balogova L., Miskovsky P., Tkacova R., Stroffekova K. (2016). Anti- and pro-apoptotic Bcl2 proteins distribution and metabolic profile in human coronary aorta endothelial cells before and after hyppdt. Cell Biochem. Biophys..

[31] Gajkowska B., Motyl T., Olszewska-Badarczuk H., Godlewski M.M. (2001). Expression of bax in cell nucleus after experimentally induced apoptosis revealed by immunogold and embedment-free electron microscopy. Cell Biol. Int..

[32] Annis M.G., Zamzami N., Zhu W.J., Penn L.Z., Kroemer G., Leber B., Andrews D.W. (2001). Endoplasmic reticulum localized Bcl-2 prevents apoptosis when redistribution of cytochrome C is a late event. Oncogene.

[33] De Brito O.M., Scorrano L. (2010). An intimate liaison: Spatial organization of the endoplasmic reticulum-mitochondria relationship. EMBO J..

[34] Osellame L.D., Blacker T.S., Duchen M.R. (2012). Cellular and molecular mechanisms of mitochondrial function. Best Pract. Res. Clin. Endocrinol. Metab..

[35] Liu J., Zhou W., Li S.S., Sun Z., Lin B.Z., Lang Y.Y., He J.Y., Cao X.H., Yan T.D., Wang L. (2008). Modulation of orphan nuclear receptor NUR77-mediated apoptotic pathway by acetylshikonin and analogues. Cancer Res..

[36] Infante S.K., Oberhauser A.F., Perez-Polo J.R. (2013). Bax phosphorylation association with nucleus and oligomerization after neonatal hypoxia-ischemia. J. Neurosci. Res..

[37] Lindenboim L., Ferrando-May E., Borner C., Stein R. (2013). Non-canonical function of bax in stress-induced nuclear protein redistribution. Cell. Mol. Life Sci..

[38] Huntosova V., Alvarez L., Bryndzova L., Nadova Z., Jancura D., Buriankova L., Bonneau S., Brault D., Miskovsky P., Sureau F. (2010). Interaction dynamics of hypericin with low-density lipoproteins and U87-mg cells. Int. J. Pharm..

[39] Huntosova V., Nadova Z., Dzurova L., Jakusova V., Sureau F., Miskovsky P. (2012). Cell death response of U87 glioma cells on hypericin photoactivation is mediated by dynamics of hypericin subcellular distribution and its aggregation in cellular organelles. Photochem. Photobiol. Sci..

[40] Wang B., Xie M., Li R., Owonikoko T.K., Ramalingam S.S., Khuri F.R., Curran W.J., Wang Y., Deng X. (2014). Role of Ku70 in deubiquitination of Mcl-1 and suppression of apoptosis. Cell Death Differ..

[41] Xie M.H., Park D., You S., Li R., Owonikoko T.K., Wang Y., Doetsch P.W., Deng X.M. (2015). Bcl2 inhibits recruitment of MRE11 complex to DNA double-strand breaks in response to high-linear energy transfer radiation. Nucleic Acids Res..

[42] Newton A.C. (2001). Protein kinase C: Structural and spatial regulation by phosphorylation, cofactors, and macromolecular interactions. Chem. Rev..

[43] Gong J.L., Holewinski R.J., van Eyk J.E., Steinberg S.F. (2016). A novel phosphorylation site at ser(130) adjacent to the pseudosubstrate domain contributes to the activation of protein kinase C-delta. Biochem. J..

[44] Nickel A., Kohlhaas M., Maack C. (2014). Mitochondrial reactive oxygen species production and elimination. J. Mol. Cell. Cardiol..

[45] Breton-Romero R., Lamas S. (2014). Hydrogen peroxide signaling in vascular endothelial cells. Redox Biol..

[46] Chong S.J.F., Low I.C.C., Pervaiz S. (2014). Mitochondrial ROS and involvement of Bcl-2 as a mitochondrial ROS regulator. Mitochondrion.

